# The yield of tuberculosis contact investigation in low- and middle-income settings: a systematic review and meta-analysis

**DOI:** 10.1186/s12879-021-06609-3

**Published:** 2021-09-27

**Authors:** Mariana Velleca, Mohsen Malekinejad, Cecily Miller, Lucia Abascal Miguel, Hailey Reeves, Philip Hopewell, Elizabeth Fair

**Affiliations:** 1 Division of Pulmonary and Critical Care Medicine, Department of Medicine, University of California, San Francisco, CA USA; 2 Institute for Global Health Sciences, University of California, San Francisco, CA USA; 3 Department of Epidemiology and Biostatistics, University of California, San Francisco, CA USA; 4 Global Tuberculosis Programme, World Health Organization, Geneva, Switzerland

**Keywords:** Contact investigation, Mycobacterium tuberculosis, Systematic Review, Tuberculosis

## Abstract

**Background:**

Contact investigation, the systematic evaluation of individuals in close contact with an infectious tuberculosis (TB) patient, is a key active case-finding strategy for global TB control. Better estimates of the yield of contact investigation can guide strategies to reduce the number of underreported and underdiagnosed TB cases, approximately three million cases per year globally. A systematic review (Prospero ID # CRD42019133380) and meta-analysis was conducted to update and enhance the estimates of the yield of TB contact investigation in low- and middle-income countries (LMIC). Pubmed, Web of Science, Embase and the WHO Global Index Medicus were searched for peer-reviewed studies (published between January 2006–April 2019); studies reporting the number of active TB or latent tuberculosis infection (LTBI) found through contact investigation were included. Pooled data were meta-analyzed using a random effects model and risk of bias was assessed.

**Results:**

Of 1,644 unique citations obtained from database searches, 110 studies met eligibility criteria for descriptive data synthesis and 95 for meta-analysis. The pooled yields of contact investigation activities for different outcomes were: secondary cases of all active TB (defined as those bacteriologically confirmed or clinically diagnosed) 2.87% (2.61–3.14, I^2^ 97.79%), bacteriologically confirmed active TB 2.04% (1.77–2.31, I^2^ 98.06%), and LTBI 43.83% (38.11–49.55, I^2^ 99.36%). Yields are interpreted as the percent of contacts screened who are diagnosed with active TB as a result of TB contact investigation activities. Pooled estimates were substantially heterogenous (I^2^ ≥ 75%).

**Conclusions:**

This study provides methodologically rigorous and up-to-date estimates for the yield of TB contact investigation activities in low- and middle-income countries (LMIC). While the data are heterogenous, these findings can inform strategic and programmatic planning for scale up of TB contact investigation activities.

## Introduction

In 2019, tuberculosis (TB) caused more than 1.4 million deaths globally, the most of any single infectious disease. During the same year there were an estimated 10 million new cases of the disease. Both the new cases and deaths occurred overwhelmingly in low- and middle-income countries (LMIC) [2–5].

To curb the TB epidemic, earlier identification and treatment of infectious individuals and their close contacts is imperative [1, 9]. Intensified case-finding efforts, including contact evaluation, can be beneficial not only to facilitate timely treatment and implementation of preventive measures, but also to address the estimated 3 million TB cases “missing” from national registries due to under-diagnosis and under-reporting [7, 9–11].

TB contact investigation, defined as the systematic evaluation of people exposed (contacts) to persons who have potentially infectious TB (index cases), is a strategy to identify additional new cases of active TB and latent TB infection (LTBI) eligible for TB preventive therapy [3, 9, 10]. Contact investigation is initiated when a new case of TB is identified [10, 13]. An index case interview is performed to obtain a list of all household and non-household contacts [10]. While procedures may vary, household visits for symptom screening of all named contacts are recommended, and those who screen positive are referred for clinical evaluation for active TB, while those who screen negative are eligible to begin preventive therapy [9, 10]. The effectiveness of contact investigation is measured by its yield, the percentage of contacts evaluated who are found to have TB [10, 18, 19].

Two older systematic reviews (Morrison et al. 2008; Fox et al. 2013) calculated the pooled yield of contact investigation in high-burden, low- and middle-income countries (LMIC) and found that among household contacts screened, 4.5% and 3.1%, respectively, had active TB (all active TB cases, bacteriologically confirmed, or clinically diagnosed) [1, 3]. These findings represent high yields for a TB control intervention considering the additional number of cases that could be found in a high TB burden setting. However, results must be analyzed with caution due to substantial heterogeneity of the studies included in both reviews [1, 3]. A secondary finding of these reviews was lack of standardized or universal definitions of the contact investigation interventions thus leading to significant heterogeneity [1, 3].

In 2012, the WHO published the first global policy guideline to assist national TB control programs with recommendations for conducting contact investigation [9]. While most of the recommendations were categorized as “strong recommendation,” most of the evidence base for these recommendations was categorized as “very low-quality evidence” due to lack of systematically collected data on contact investigation activities in LMIC [22]. A central hypothesis of the expanded systematic review described here is that the number of activities in low-resource, high TB burden settings has increased significantly and have been conducted with a more systematic approach, thus warranting an update to the literature.

Findings on the effectiveness of TB contact investigation in LMIC, defined as the pooled yield of secondary TB cases and LTBI cases detected among contacts screened, are presented by updating the systematic review by Morrison et al. published in 2008 [1]. Additionally, the impact of the publication of the WHO TB Contact Investigation Policy Guidelines [9] in 2012 on the number of studies and on the yield of TB contact investigation was assessed.

## Methods

A comprehensive protocol to inform our systematic review study was developed and registered. (Prospero ID # CRD42019133380).

### Search strategy and eligibility criteria

Pubmed, Web of Science, Embase and The World Health Organization Global Index Medicus were searched for peer-reviewed literature to identify studies published between January 2006 and April 2019 which reported the number of active TB cases or persons with LTBI found through contact investigation of close contacts of infectious TB cases in LMIC. See Additional file 1: Table S1 for search dates, keywords used, and number of citations found for each database and Additional file 2: Table S2 for the key-definitions that were used for this systematic review.

Studies which reported data on pulmonary and extrapulmonary TB index cases were included. There were no age restrictions for index cases nor for TB contacts. Data were analyzed considering the following age groups for TB contacts: < 5 years; 5–14 years; < 15 years; ≥ 15 years.

Studies that reported the number of secondary active TB cases (clinically diagnosed or bacteriologically confirmed) or LTBI cases as the numerator, and the total number of contacts screened as the denominator, to calculate the yield of contact investigation were included. Study designs included: prospective cohort studies, cross-sectional studies, retrospective studies, cluster-randomized studies and longitudinal cohort studies.

Retrospective studies that did not clearly distinguish between co-prevalent cases (individuals with TB diagnosed within three months of the index case diagnosis) and incident cases (diagnosed > 3 months after the index case diagnosis) as well as other systematic reviews were excluded.

There were no restrictions on eligibility by language of publication. See Additional file 3: Table S3 for the complete list of exclusion criteria.

### Selection of studies & data extraction process

All search results were imported into the bibliographic citation manager EndNote X9.2 [23] to remove duplicate records. Remaining citations were exported to the web application Rayyan QCRI [24] for title and abstract screening. First author (MV) reviewed titles/abstracts of all citations and two co-authors (HR and LA) reviewed (50% each) titles/abstracts of citations excluded by MV. Next, MV reviewed full-text of included citations and two co-authors (HR and LA) reviewed a 10% random sample of full-texts. Any discordance was resolved by consensus with two senior authors (EF and CM) as mediators.

A web-based data extraction form using RedCap software was developed, and pilot tested prior to the data extraction phase [25, 26]. MV extracted data for all eligible studies, and two co-authors (HR and LA) reviewed a 10% random sample of extracted data. The level of discordance between MV and HR/LA was 2.1%, resolved by consensus with two senior authors (EF and CM) as mediators.

For publications presenting data of the same contact investigation, the most recent and comprehensive publication was included for data extraction and the other publications referenced for missing information if needed. Online supplementary appendices of included studies were reviewed for relevant information.

A validated tool for the assessment of risk of bias in prevalence studies was adapted for this systematic review [27]. See Additional file 4: File S1 for the adapted risk of bias tool and a table comparing the questions of the original tool to the adapted one.

### Data synthesis

The unit of analysis was the close contacts of active TB cases. The common metric of interest was the yield of contact investigation, calculated by: the number of secondary cases of TB found through contact investigation (active TB or LTBI) divided by the number of contacts screened.

Metaprop command in Stata/MP 15.1 [28] was used to calculate the pooled proportions and 95% confidence intervals for the yield of contact investigation and produced the forest plots using random effects model [29,1,3]. Statistical heterogeneity was assessed using the chi-squared test and calculating the I^2^,^2^ threshold by Cochrane Handbook was used for their interpretation (considerable heterogeneity I^2^ ≥ 75%) [30].

Subgroup analysis was conducted to calculate the pooled yield of contact investigation stratified by: WHO geographic region [34]; World Bank income classification [33]; age group (< 5y; 5-14y; < 15y; ≥ 15y); contacts with positive HIV status; contacts of multi drug resistant (MDR)-TB index cases; smear status of index case; location of the contact investigation (community-based or clinic-based); and by start date of data collection (before or after the publication of the WHO TB Contact Investigation Policy Guidelines [9] in 2012) The overall analysis and all sub-group analyses were performed separately for active TB and then for LTBI.

Sensitivity analysis to assess the effect of risk of bias on the results was performed by removing studies classified as moderate and high risk of bias from the analysis. Also assessed was the effect of removing the top 5% and bottom 5% outliers from the analysis for the pooled yields of all active TB, confirmed active TB and LTBI. Additional analysis was performed by excluding studies with restrictions for the target population of contacts based on age, or restrictions based on HIV status and drug susceptibility for the target population of index cases.

## Results

### Descriptive results

**Fig. 1:**
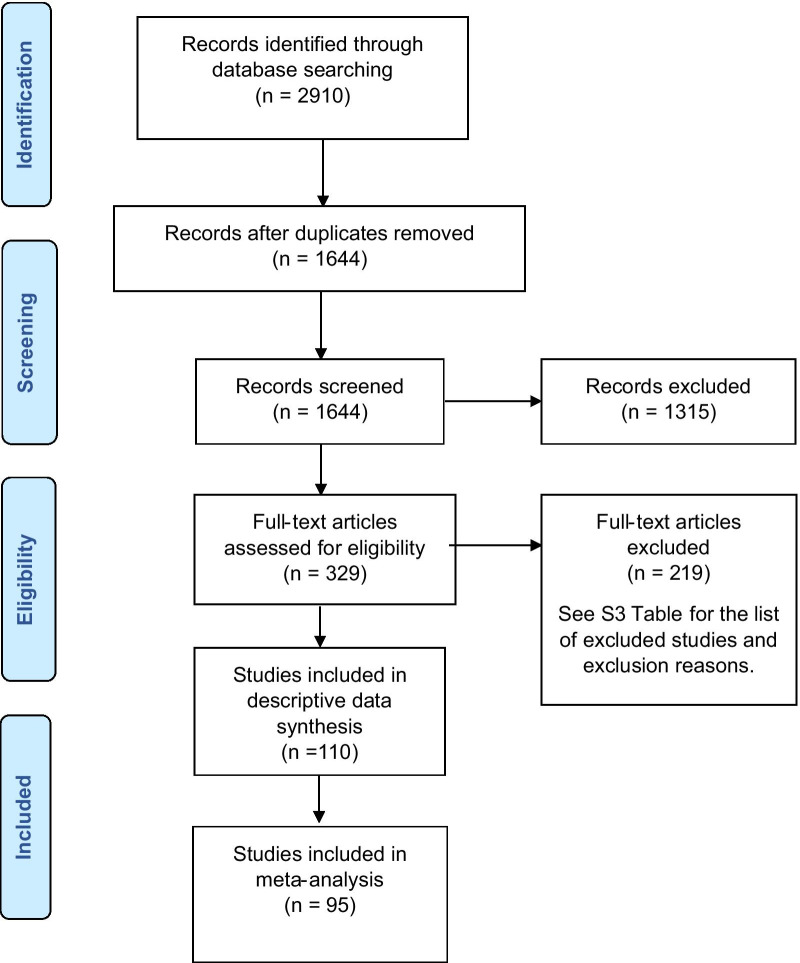
Flow diagram for study selection

The Preferred Reporting Items for Systematic Reviews and Meta-Analyses (PRISMA) checklist was used to guide reporting [35]. The study search and selection process is shown in Fig. 1.

Through database search, 1,644 unique citations were identified; 110 studies were classified as eligible for data extraction. Additional file 5: Table S4 provides the list of citations included in this systematic review.

Included studies were conducted in 42 different countries, the majority conducted in Africa (39%) and Southeast Asia (16%), based on the WHO geographical region classification [34]. There was a relatively even balance in the distribution of the eligible publications by World Bank income classification [33], with 29% of the studies conducted in low-income countries, 35% in lower middle-income countries, and 35% in upper middle-income countries.

**Fig. 2:**
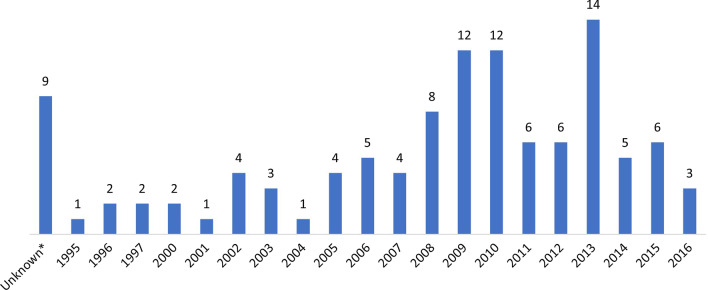
Distribution of studies per year based on the start date of data collection (n = 110). *: there was no information on the start date of data collection, but the publication of these studies was before September 2012

Seventy-nine (72%) of the included studies started data collection before the publication of the WHO TB Contact Investigation Policy Guidelines [9,

Figure 2 shows the distribution of studies per year based on start date of data collection. The year with the highest number of studies starting data collection was 2013 (14 studies).

The median number of index cases per study was 254.5 (range 30–47,021, IQR 119.5–729.5), the median number of contacts screened in each study for active TB was 665 (range 31–33,631, IQR 252–2585) and the median number of contacts screened in each study for LTBI was 345 (range 28–12,648, IQR 195–1064).

**Fig. 3:**
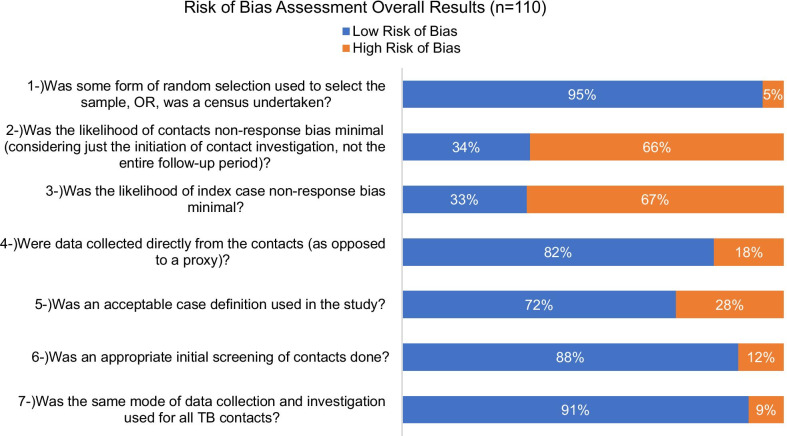
Overall results of the risk of bias assessment (n = 110)

Forty studies presented data for child contacts under five years of age (< 5y), involving 17,556 children < 5y screened for secondary active TB. Twenty-one studies reported a total of 6,479 children < 5y started on Isoniazid Preventive Therapy (IPT). Figure 3 shows the overall results of the risk of bias assessment. See Additional file 6: Table S5 for the complete data on risk of bias for each individual study.

### Meta-analysis

**Fig. 4:**
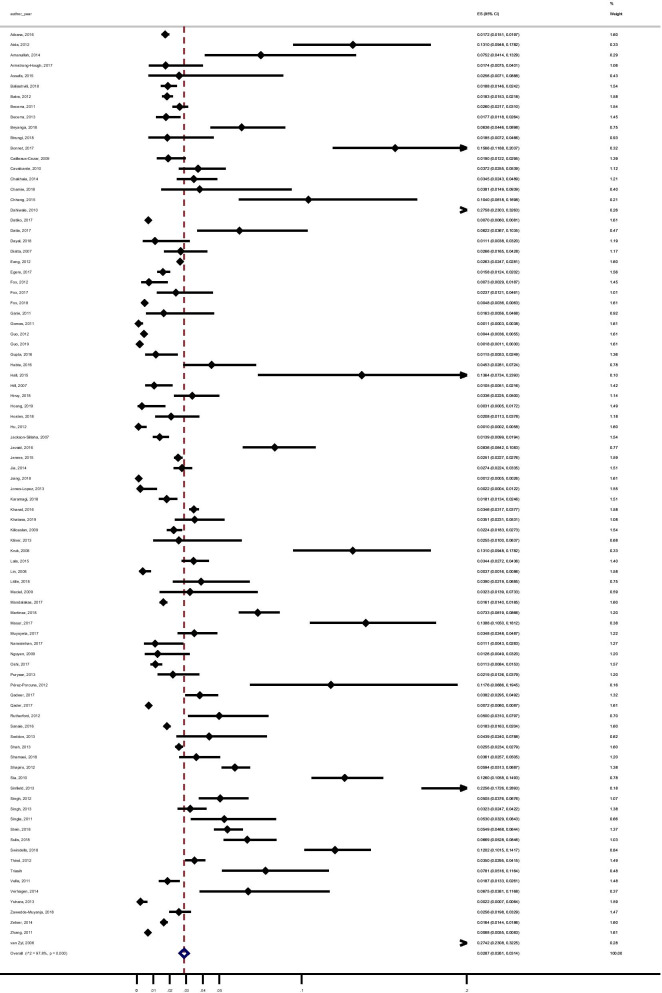
Forest plot of the yield of contact investigations for all active tuberculosis (confirmed and clinically/ radiologically diagnosed)

**Fig. 5:**
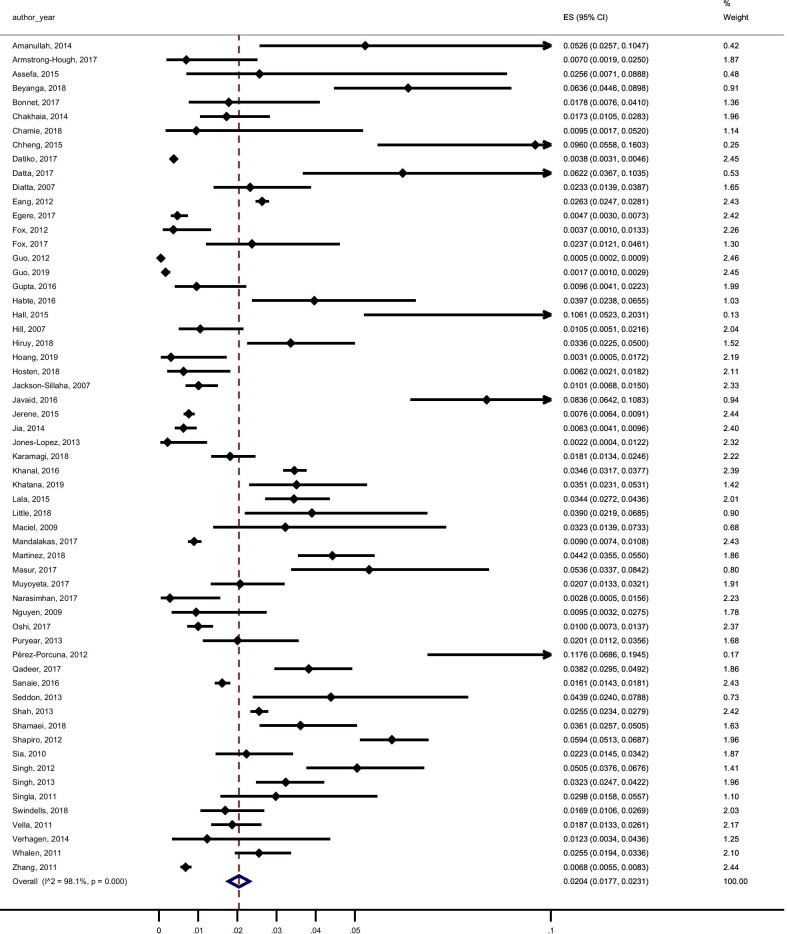
Forest plot of the yield of contact investigations for confirmed tuberculosis

**Fig. 6:**
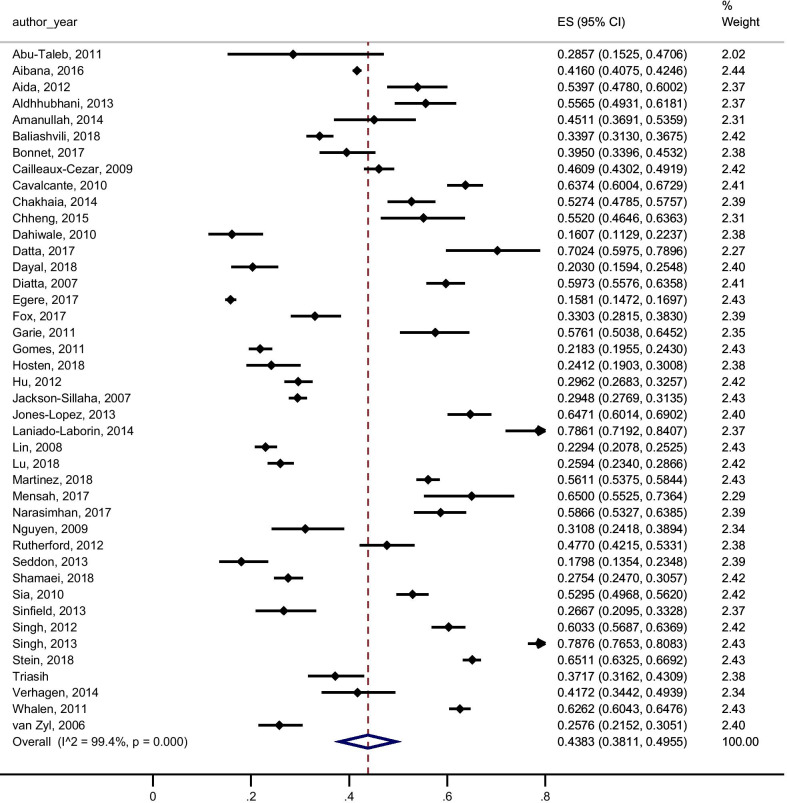
Forest plot of the yield of contact investigations for LTBI

Among the included studies, 95 reported data to calculate the pooled yield of contact investigation for secondary cases of active TB (all active TB), 59 studies provided data to calculate the pooled yield of contact investigation for secondary cases of confirmed active TB and 42 studies reported data for the pooled yield of contact investigation for LTBI. The results with a 95% confidence interval and I^2^ for heterogeneity were: 2.87% (2.61–3.14, I^2^ 97.79%) for all active TB (Fig. 4), 2.04% (1.77–2.31, I^2^ 98.06%) for confirmed active TB (Fig. 5) and 43.83% (38.11–49.55, I^2^ 99.36%) for LTBI (Fig. 6).

### Subgroup analyses

Tables 1 and 2 summarize the results obtained for subgroup analyses. There was considerable heterogeneity (I^2^ ≥ 75%) in all subgroups.

**Table 1:** Pooled yields among subgroups for all active TB, confirmed active TB, and LTBI

Subgroups	n	Pooled % yield for all active TB (95% confidence interval)	n	Pooled % yield for confirmed active TB (95% confidence interval)	n	Pooled % yield for LTBI (95% confidence interval)
WHO Geographic regions						
AFR	37	3.43% (2.91–3.94, I^2^ 97.09%)	27	1.97% (1.62–2.31, I^2^ 94.58%)	15	44.14% (32.52–55.76, I^2^ 99.62%)
AMR	12	2.68% (1.95–3.41, I^2^ 94.70%)	5	4.77% (2.02–7.51, I^2^ 78.37)	6	56.74% (45.78–67.69, I^2^ 98.31%)
EMR	10	3.11% (2.33–3.90, I^2^ 97.29%)	7	2.98% (2.13–3.82, I^2^ 94.48%)	6	39.43% (27.23–51.62, I^2^ 95.94%)
EUR	3	2.30% (1.69–2.91)*	1	1.73% (1.05–2.83)*	2	38.44% (36.06–40.82)*
SEAR	12	4.81% (3.42–6.21, I^2^ 93.53%)	8	2.93% (1.59–4.27, I^2^ 94.77%)	7	45.63% (26.66–64.60, I^2^ 99.31%)
WPR	14	1.17% (0.75–1.59, I^2^ 98.58%)	10	0.94% (0.42–1.46, I^2^ 99.04%)	6	32.58% (23.38–41.79, I^2^ 97.90%)
World Bank income classification						
Low-income	28	3.00% (2.49–3.52, I^2^ 97.70%)	21	1.97% (1.5–2.44, I^2^ 97.04%)	13	46.89% (34.23–59.54, I^2^ 99.67%)
Lower middle-income	30	3.29% (2.74–3.84, I^2^ 96.83%)	21	2.23% (1.69–2.76, I^2^ 95.62%)	16	44.85% (34.57–55.13, I^2^ 98.79%)
Upper middle-income	30	2.22% (1.86–2.58, I^2^ 97.52%)	16	1.78% (1.40–2.16, I^2^ 96.73%)	13	39.34% (32.15–46.54, I^2^ 98.73%)
Year started data collection						
Pre WHO guideline	63	2.77% (2.47–3.08, I^2^ 97.97%)	38	1.99% (1.67%-2.31%, I^2^ 98.34%)	39	45.22% (39.25–51.19, I^2^ 99.40%)
Post WHO guideline	25	3.08% (2.49–3.68, I^2^ 96.33%)	20	2.10% (1.57–2.64, I^2^ 95.09%)	3	25.80% (18.19–33.40)*
HIV status						
Positive contacts	9	8.95% (5.49–12.41, I^2^ 80.53%)	5	6.71% (2.15–11.27, I^2^ 89.11%)	0	N/A
Sputum smear status of index case						
Positive sputum smear index	5	8.30% (3.88–12.73, I^2^ 85.36%)	N/A	N/A	N/A	N/A
MDR-TB						
Contacts of MDR-TB	13	4.69% (3.26–6.13, I^2^ 94.30%)	10	3.43% (2.05–4.82, I^2^ 90.28%)	7	37.53% (24.22–50.84, I^2^ 95.99%)
Location of contact investigation						
Community-based	36	2.34% (1.96–2.72, I^2^ 97.89%)	28	2.14% (1.72–2.55, I^2^ 97.96%)	17	46.67% (36.86–56.49, I^2^ 99.64%)
Clinic-based	38	3.44% (2.96–3.93, I^2^ 97.93%)	24	1.90% (1.37–2.43, I^2^ 98.03%)	20	40.15% (32.81–47.49, I^2^ 98.18%)

*AFR* Africa, *AMR* Americas, *EMR* Eastern Mediterranean, *EUR* Europe, *SEAR* South-East Asia, *WPR* Western Pacific

*Due to small study size it was not possible to assess heterogeneity

**Table 2:** Pooled data for all active TB and confirmed active TB among contacts, by age group

Subgroups	Studies (n)	Pooled % yield (95% confidence interval)
All active TB		
< 5y	32	6.84% (5.56–8.11, I^2^ 95.95%)
5–14y	11	3.13% (2.11–4.16, I^2^ 85.81%)
< 15y	28	3.59% (2.72–4.46, I^2^ 95.30%)
≥ 15y	26	3.69% (3.0–4.37, I^2^ 91.81%)
Confirmed active TB		
< 5y	10	0.73% (0.24–1.22, I^2^ 79.03%)
5–14y	5	3.43% (1.02–5.85, I^2^ 76.27%)
< 15y	15	1.65% (1.03–2.27, I^2^ 86.85%)
≥ 15y	20	3.22% (2.27–4.17, I^2^ 91.23%)

Examining 95% confidence intervals (CI), a lower pooled estimate in the Western Pacific region was observed for all active TB (1.17%, 95%CI: 0.75–1.59, I^2^ 98.58%) and for confirmed active TB (0.94%, 95%CI: 0.42–1.46, I^2^ 99.04%) when compared to the other regions. For LTBI the point estimates and 95% CI were considerably overlapping for all regions.

The upper middle income World Bank group had a lower pooled estimate (2.22%, 95%CI: 1.86–2.58, I^2^ 97.52%) without an overlapping 95% confidence interval compared to the lower middle-income group (3.29%, 95%CI: 2.74–3.84, I^2^ 96.83%) for all active TB. For both confirmed active TB and LTBI the point estimates and 95% confidence intervals were considerably overlapping for all the income groups.

Regarding age subgroups, a statistically significant higher pooled yield was found for the group of children under five years of age for all active TB (6.84%, 95%CI: 5.56–8.11, I^2^ 95.95%). Statistically significant lower pooled yields were found for confirmed active TB when compared to all active TB for the subgroups of children < 5y (0.73%, 95% CI: 0.24–1.22, I^2^ 79.03%) and < 15y (1.65%, 95%CI: 1.03–2.27, I^2^ 86.85%).

Clinic-based contact investigation yielded a higher number of TB cases (3.44%, 95%CI: 2.96–3.93, I^2^ 97.93%) when compared to community-based contact investigation (2.34%, 95%CI: 1.96–2.72, I^2^ 97.89%).

,^2^,^2^,^2^,^2^,^2^ 85.36% for all active TB) when compared to the overall pooled yields of contact investigation. No statistically significant differences in the estimates were found by start date of data collection (before or after the publication of the WHO TB Contact Investigation Policy Guidelines [9] in 2012).

### Sensitivity analysis

No significant changes on the estimates for the pooled yields of all active TB, confirmed active TB and LTBI were observed when the analysis was repeated after removing the top 5% and bottom 5% outliers, nor after removing studies with restrictions for the target population of contacts based on age, or restrictions based on HIV status and drug susceptibility for the target population of index cases (See Additional file 7: File S2 for details).

Additional sensitivity analysis was carried out by calculating the pooled yields without the studies classified as high risk of bias, and without the studies classified as either moderate or high risk of bias (See Additional file 7: File S2 for details). A slightly lower pooled yield was observed when including just studies classified as low risk of bias for all active TB: 2.01% (1.47–2.55, I^2^ 98.13%).

## Discussion

The systematic review presented here provides a timely update on the literature on the yield of TB contact investigation and more robust evidence of the contribution of contact investigation activities to TB case detection in LMIC. The previous systematic review published in 2008 [1], included 41 publications covering the period from 1955 to 2005 (50 years), while the current review included 110 studies in a period of just 13 years, indicating the number of TB contact investigation publications has increased substantially over the years as hypothesized. With the persistent estimate of 3–4 million “missing” TB cases reported globally over the last 5 years, contact investigation as an imperative active case finding strategy is being prioritize in LMIC and high TB burden settings.

Although the number of studies starting data collection after the publication of the WHO TB Contact Investigation Policy Guideline in 2012 is significantly smaller (28 studies) than the number of studies starting data collection before the guideline (79 studies), the publications included in this review covered studies which started data collection from 1995 to 2016, thus the period prior to the publication of the guideline was longer (Fig. 2). The year with the highest number of studies starting data collection was 2013, which indicates a potential boosting impact of the guideline on contact investigation activities (Fig. 2). On average there was a gap of five years between the start date of data collection and the publication date of a study, thus we could expect an increase on the number of studies starting data collection after 2013, which will be published over the next years, with an exception for the years during the COVID-19 pandemic, which has disrupted TB investigation activities worldwide.

,^1^,^3^,

An important consideration is, while the use of the yield of contact investigation is a well-established statistic, both the numerator and denominator can be impacted by how the activities are implemented. For example, use of rapid diagnostics or early initiation of therapy for active TB patients may drive the yield down because transmission time may be decreased, while targeted screening may increase the yield compared with comprehensive screening of larger population of contacts. In this review, the median number of contacts found and screened in each study for active TB was 665 (range 31–33,631, IQR 252–2585) while the median number reported by Morrison et al. in 2008 was 523.5 (range 56–3046, IQR 286.25–1012.25).

In the subgroup analyses (Tables 1 and 2), high heterogeneity was found, therefore the findings should be interpreted with caution. The higher pooled yield for clinic-based contact investigation when compared to community-based contact investigation for all active TB was expected, considering that a higher number of symptomatic contacts is found with this passive strategy. The differences between the estimates for all active TB and confirmed active TB across age groups can be explained by the fact that standard practice often does not include confirmation of TB diagnosis in children.

Many studies did not report data regarding smear positivity of the index case, drug susceptibility of the index case nor HIV status of the contacts, thus estimates presented for these subgroups might not be accurate. Despite the small number of studies included in the analysis, the results indicate a potentially high yield for all active TB among these risk groups. This finding has important implications for countries when grappling with decisions about who to prioritize for contact investigation activities, and for infection control activities considering the potentially worse outcomes related to undiagnosed TB in these groups, particularly in contacts who are PLHIV and contacts of MDR-TB.

The higher pooled yields found for all active TB for children under five years of age and for PLHIV, when compared to the overall pooled yield, highlight the importance of establishing and/or sustaining policies for TB preventive therapy in these countries.

Removing outliers for the sensitivity analysis did not lead to a meaningful effect on the estimates. When including only studies classified as low risk of bias, the estimates changed significantly (Additional file 7: File S2). However, the number of studies included in this analysis was small.

Although the risk of publication bias cannot be excluded, a formal assessment using funnel plots and regression asymmetry was not conducted because the validity and utility of these methods in meta-analyses of proportions is uncertain [32].

The adapted risk of bias tool used was helpful in assessing bias in the studies by focusing in aspects such as contacts response rates, the definitions used, the sampling method used, source of data collection and standardization of procedures. The high percentage of studies classified as high risk of bias for the domain of non-response bias (see Additional file 6: Table S5) can be explained by the fact that, as stated in many studies, the investigators could not screen all the listed contacts due to absence, travelling or refusal to participate. In many studies where clinic-based contact investigation was performed, only a small percentage of contacts presented to the clinic for evaluation, and in the case of retrospective studies the number of contacts listed compared to the number that attended the clinic for assessment is unknown. Index case response rates were affected by refusal to participate or by not referring contacts for evaluation.

Further studies are needed with review of the broader literature, including contact investigation studies conducted in high income countries, as well as stratified analyses by screening and diagnostic approach used to determine impact of the screening algorithm on yield of prevalent cases found. Analyses of the timing of contact investigation to determine optimal follow up time of the intervention is also an important area of research where the data remains sparse.

## Limitations

### Limitations of primary studies

This study had several limitations. In addition to substantial limitations from the heterogeneity of the studies included, the number of contacts screened per index case was not captured by this systematic review, and this could be an important information regarding the systematization of contact investigation activities. Although studies with contacts recently exposed to TB index cases (co-prevalent cases) were targeted, some incident and co-prevalent cases may have been merged in the estimates because the included studies used different time-periods for classification of baseline.

### Limitations of the review

Limitations that may have affected the results include using a single reviewer for full-text screening and data extraction, with only a percentage verified by co-authors, as well as the decision not to search grey literature and not to include programmatic data on TB contact investigation activities.

## Conclusion

Despite substantial heterogeneity, this systematic review contributes important additional evidence for the effectiveness and impact of contact investigation in LMIC. Continued observations over the following years will be required to assess whether the number of contact investigation activities have increased significantly after the publication of the WHO Contact Investigation Guideline in 2012, whether the activities have been conducted more systematically, and ultimately what contribution to and impact on TB case finding contact investigation has.

## Supplementary Information

**Additional file 1: Table S1.** Mesh-terms and keywords used for each database, search date and the number of citations found.

**Additional file 2: Table S2.** Key-definitions that were used for this systematic review.

**Additional file 3: Table S3.** List of excluded studies and exclusion reasons.

**Additional file 4: File S1. **Adapted Risk of Bias Assessment Tool.

**Additional file 5: Table S4.** List of the 110 citations included in this systematic review.

**Additional file 6: Table S5. **Data on Risk of Bias for each study.

**Additional file 7: File S2.** Sensitivity Analysis.

## Data Availability

The datasets used and/or analyzed during the current study are available from the corresponding author on reasonable request.

## Abbreviations

TB: Tuberculosis
LMIC: Low- and middle-income countries
LTBI: Latent tuberculosis infection
MDR: Multi drug resistant
WHO: World Health Organization
PRISMA: Preferred Reporting Items for Systematic Reviews and Meta-Analyses
IPT: Isoniazid Preventive Therapy
CI: Confidence intervals
PLHIV: People living with HIV
