# Supplementary material for: The yield of tuberculosis contact investigation in low- and middle-income settings: a systematic review and meta-analysis
Source: BMC Infect Dis. 2021 Sep 27;21:1011. doi: 10.1186/s12879-021-06609-3 (PMC8474777; doi:10.1186/s12879-021-06609-3)
Supplement: Supplementary file 1 — Additional file 1: Table S1. Mesh-terms and keywords used for each database, search date and the number of citations found. [file 12879_2021_6609_MOESM1_ESM.pdf]

S1 Table. Mesh-terms and keywords used for each database, search date and the number of citations found.

Database	Search date	Search Strategy	Studies found	Duplicates	Final
**Pubmed**	4/23/2019	(((((“Tuberculosis”[MeSH] OR “Mycobacterium tuberculosis”[MeSH] OR “tuberculosis”[TI])))) AND (("contact investigation" OR "contact screening" OR "contact examination" OR "household screening" OR "contact screen" OR "case find" OR "case finding" OR "case detection" OR (cluster* AND analys*) OR Contact Tracing[Mesh])) AND (("close family contact" OR "family contact" OR "close contact" OR "household" OR "tuberculosis contact" OR "childhood contact" OR "adult contact" OR "contact")))	1100	18	1082
**Web of Science**	4/23/2019	TS=(((((“Tuberculosis”[MeSH] OR “Mycobacterium tuberculosis”[MeSH] OR “tuberculosis”)) AND ("contact investigation" OR "contact screening" OR "contact examination" OR "household screening" OR "contact screen" OR "case find" OR "case finding" OR "case detection" OR (cluster*AND analys*) OR Contact Tracing[Mesh])) AND ("close family contact" OR "family contact" OR "close contact" OR "tuberculosis contact" OR "household" OR "childhood contact" OR "adult contact" OR "contact")))	494	489	5
**Embase**	4/23/2019	('tuberculosis'/exp OR 'mycobacterium tuberculosis' OR tuberculosis OR 'tuberculosis transmission'/exp OR 'tuberculosis transmission') AND ('contact investigation' OR 'contact screening' OR 'contact examination'/exp OR 'contact examination' OR 'household screening' OR 'contact screen' OR 'case find' OR 'case finding'/exp OR 'case finding' OR 'case detection' OR (cluster* AND analys*)) AND ('close family contact' OR 'family contact' OR 'close contact' OR 'household contact'/exp OR 'household contact' OR 'household infection' OR 'tuberculosis contact' OR 'household transmission' OR 'household cluster' OR 'childhood contact' OR 'adult contact' OR 'attack contact' OR 'attack rate contact' OR 'contact'/exp OR 'contact') AND [2006-2019]/py	1250	729	521
**WHO Global Index Medicus**	4/24/2019	"tuberculosis contact investigation" OR "tuberculosis contact tracing"	66	30	36
		**TOTAL**	**2910**	**1266**	**1644**
