# Supplementary material for: The yield of tuberculosis contact investigation in low- and middle-income settings: a systematic review and meta-analysis
Source: BMC Infect Dis. 2021 Sep 27;21:1011. doi: 10.1186/s12879-021-06609-3 (PMC8474777; doi:10.1186/s12879-021-06609-3)
Supplement: Supplementary file 2 — Additional file 2: Table S2. Key-definitions that were used for this systematic review. [file 12879_2021_6609_MOESM2_ESM.pdf]

S2 Table. Key-definitions that were used for this systematic review.

Country income classification	The World Bank income classification criteria will be used to classify the countries.[34]
**Index case**	The first patient of active TB identified, around whom the investigation is centered.[9,10]
**Close contact**	A person sharing an enclosed space with the index case, in the household or not, for extended periods during the 3 months before the commencement of the TB episode.[19]
**Household contact**	A person living in the same household of the index case. [3]
**LTBI**	A person with TST induration greater than 10 mm (or the publication cut-off) in whom active TB has been excluded.[1] OR a person with a positive interferon gamma release assay (IGRA) result based on the publication cut-off.[3]
**Yield of contact investigation (%)**	The number of secondary cases of active TB or LTBI found divided by the number of contacts screened.[19]
**Contact with confirmed active TB**	A contact with smear or culture positive for *M. tuberculosis.*[1]
**Subgroup analysis by geographic region**	Studies will be stratified by the six World Health Organization regions. [3,35]
