# Supplementary material for: The yield of tuberculosis contact investigation in low- and middle-income settings: a systematic review and meta-analysis
Source: BMC Infect Dis. 2021 Sep 27;21:1011. doi: 10.1186/s12879-021-06609-3 (PMC8474777; doi:10.1186/s12879-021-06609-3)
Supplement: Supplementary file 3 — Additional file 3: Table S3. List of excluded studies and exclusion reasons. [file 12879_2021_6609_MOESM3_ESM.pdf]

**S3 Tables - Excluded Studies**

**S3.1 Table. List of exclusion reasons and number of excluded studies at full-text level.**

| Exclusion number | Description | Total excluded studies |
|---|---|---|
| **1** | High-income country | 19 |
| **2** | Abstracts, editorials, correspondence | 52 |
| **3** | Case studies or outbreak reports | 11 |
| **4** | Only contacts with active TB (no data to calculate yield) | 4 |
| **5** | Total number of contacts screened not reported (no data to calculate yield) | 2 |
| **6** | No data do calculate yield (other) | 29 |
| **7** | No systematic evaluation of contacts | 26 |
| **8** | Retrospective study with no data for co-prevalent cases | 21 |
| **9** | Duplicated data | 17 |
| **10** | Other | 27 |
| **11** | No contact investigation conducted | 12 |

**S3.2 Table. List of excluded studies at full-text level and reasons for exclusion.**

| Reference ID | Author, Year | Exclusion Reason | Comments for "Other" |
|---|---|---|---|
| **195** | Aissa, 2008 | 1 | |
| **9** | Andre, 2007 | 1 | |
| **12** | Atamna, 2009 | 1 | |
| **27** | Barcellini, 2016 | 1 | |
| **197** | Bari, 2007 | 1 | |
| **162** | Chao, 2016 | 1 | |
| **50** | Chou, 2015 | 1 | |
| **159** | Duarte, 2012 | 1 | |
| **201** | Lee, 2015 | 1 | |
| **143** | Leung, 2013 | 1 | |
| **225** | Martinez Lacasa, 2015 | 1 | |
| **32** | Molton, 2019 | 1 | |
| **67** | Pan, 2016 | 1 | |
| **164** | Piccini, 2017 | 1 | |
| **183** | Salinas, 2006 | 1 | |
| **70** | Uzorka, 2018 | 1 | |
| **218** | Wang, 2012 | 1 | |
| **41** | Yoo, 2016 | 1 | |
| **203** | Zellweger, 2015 | 1 | |
| **282** | Aldeco, 2011 | 2 | |
| **283** | Altet, 2012 | 2 | |
| **284** | Altet, 2013 | 2 | |
| **160** | Atif, 2013 | 2 | |
| **285** | Auld, 2016 | 2 | |
| **286** | Benhalla, 2011 | 2 | |
| **287** | Benson, 2016 | 2 | |
| **288** | Bothamley, 2017 | 2 | |

| | | |
|---|---|---|
| **33** | Buu, 2010 | 2 |
| **37** | Campbell, 2006 | 2 |
| **319** | Chelnokova, 2010 | 2 |
| **290** | Cirri, 2016 | 2 |
| **199** | Do Nascimento Maia, 2016 | 2 |
| **232** | Duenas, 2009 | 2 |
| **145** | Enuechie, 2017 | 2 |
| **294** | Fox, 2012 | 2 |
| **293** | Fox, 2013 | 2 |
| **292** | Fox, 2017 | 2 |
| **71** | Fox, 2018 | 2 |
| **104** | Fox, 2019 | 2 |
| **40** | Frieden, 2016 | 2 |
| **153** | Hawkridge, 2007 | 2 |
| **200** | Hersch, 2017 | 2 |
| **113** | Jain, 2018 | 2 |
| **26** | Jeena, 2015 | 2 |
| **297** | Kaguthi, 2010 | 2 |
| **298** | Kartasasmita, 2011 | 2 |
| **299** | Khan, 2016 | 2 |
| **300** | Kimaro, 2018 | 2 |
| **301** | Kurhasani, 2013 | 2 |
| **302** | Llacer, 2010 | 2 |
| **36** | Lorent, 2014 | 2 |
| **127** | Martinez, 2018 | 2 |
| **185** | Nguenha, 2018 | 2 |
| **304** | Olivianto, 2012 | 2 |
| **305** | Paul, 2014 | 2 |
| **306** | Perez-Escolano, 2009 | 2 |
| **307** | Qayyum, 2013 | 2 |
| **308** | Salazar-Austin, 2017 | 2 |
| **309** | Sargazi, 2015 | 2 |

| | | |
|---|---|---|
| **310** | Scully, 2009 | 2 |
| **25** | Shah, 2010 | 2 |
| **311** | Shah, 2011 | 2 |
| **39** | Shrivastava, 2014 | 2 |
| **312** | Simonovska, 2013 | 2 |
| **313** | Triasih, 2012 | 2 |
| **314** | Triasih, 2013 | 2 |
| **315** | Tumusiime, 2017 | 2 |
| **316** | Vilc, 2013 | 2 |
| **114** | Wardhana, 2016 | 2 |
| **112** | Wiwanitkit, 2012 | 2 |
| **79** | Yates, 2018 | 2 |
| **28** | Baghaie, 2012 | 3 |
| **20** | Bamrah, 2014 | 3 |
| **289** | Branch, 2013 | 3 |
| **17** | CDC, 2009 | 3 |
| **256** | Fred, 2010 | 3 |
| **60** | Huang, 2016 | 3 |
| **24** | Ilic, 2019 | 3 |
| **239** | Pan, 2018 | 3 |
| **85** | Pan, 2019 | 3 |
| **61** | Popovici, 2018 | 3 |
| **149** | Warrington, 2010 | 3 |
| **58** | Gonzalez Diaz, 2015 | 4 |
| **163** | Marais, 2006 | 4 |
| **173** | Seda-Geylani, 2012 | 4 |
| **111** | Wang, 2014 | 4 |
| **44** | Hernández-Garduño, 2015 | 5 |
| **22** | Morishita, 2016 | 5 |
| **156** | Adetifa, 2010 | 6 |
| **16** | Armstrong-Hough, 2018 | 6 |
| **19** | Belgaumkar, 2018 | 6 |

| | | |
|---|---|---|
| **129** | Buchwald, 2014 | 6 |
| **57** | Chabala, 2017 | 6 |
| **166** | Chauhan, 2013 | 6 |
| **78** | Correia Sacchi, 2018 | 6 |
| **66** | Crampin, 2009 | 6 |
| **146** | García de la Rosa, 2014 | 6 |
| **97** | Ha, 2016 | 6 |
| **155** | Hartwig, 2009 | 6 |
| **170** | Hector, 2017 | 6 |
| **64** | Hill, 2006 | 6 |
| **220** | Jones-Lopez, 2014 | 6 |
| **157** | Kabongo, 2010 | 6 |
| **99** | Kebede, 2018 | 6 |
| **88** | Khan, 2017 | 6 |
| **209** | Kigozi, 2018 | 6 |
| **119** | Kone, 2016 | 6 |
| **92** | Monárrez-Espino, 2014 | 6 |
| **280** | Munir, 2017 | 6 |
| **31** | Ottmani, 2009 | 6 |
| **86** | Pinto de Oliveira, 2013 | 6 |
| **188** | Shakak, 2013 | 6 |
| **277** | Shalabi, 2009 | 6 |
| **91** | Shanaube, 2011 | 6 |
| **38** | Uwimana, 2012 | 6 |
| **324** | Xie, 2016 | 6 |
| **108** | Zunic, 2012 | 6 |
| **2** | Adetifa, 2017 | 7 |
| **5** | Albanese, 2016 | 7 |
| **63** | Ayles, 2013 | 7 |
| **47** | Bai, 2008 | 7 |
| **198** | Bayhan, 2015 | 7 |
| **326** | Berraies, 2016 | 7 |

| | | |
|---|---|---|
| **130** | Calver, 2010 | 7 |
| **318** | Chelnokova, 2010 | 7 |
| **106** | Chiang, 2015 | 7 |
| **231** | Dundar, 2019 | 7 |
| **126** | Fortunato, 2011 | 7 |
| **169** | Gebregergs, 2015 | 7 |
| **18** | ICMR, 2011 | 7 |
| **59** | Kilale, 2016 | 7 |
| **14** | Koenig, 2015 | 7 |
| **148** | Mandalakas, 2012 | 7 |
| **196** | Mandalakas, 2015 | 7 |
| **103** | Rajan, 2017 | 7 |
| **229** | Ranganath, 2018 | 7 |
| **21** | Rivera, 2017 | 7 |
| **98** | Singh, 2017 | 7 |
| **252** | Wiseman, 2015 | 7 |
| **96** | Wysocki, 2016 | 7 |
| **122** | Yirgu, 2017 | 7 |
| **110** | Zhang, 2015 | 7 |
| **228** | Zhang, 2019 | 7 |
| **321** | Abakay, 2006 | 8 |
| **236** | Chatla, 2018 | 8 |
| **51** | Coprada, 2016 | 8 |
| **221** | Dair García de la Rosa, 2014 | 8 |
| **233** | de Lima, 2013 | 8 |
| **176** | Fatima, 2016 | 8 |
| **226** | Gazetta, 2006 | 8 |
| **147** | Grandjean, 2011 | 8 |
| **177** | Joshi, 2015 | 8 |
| **230** | Khalilzadeh, 2006 | 8 |
| **223** | Khazaei, 2018 | 8 |
| **323** | Kilicaslan, 2006 | 8 |

| | | | |
|---|---|---|---|
| **158** | Kisa, 2016 | 8 | |
| **172** | Loredo, 2014 | 8 | |
| **247** | Nair, 2016 | 8 | |
| **234** | Naowarat, 2018 | 8 | |
| **242** | Ohene, 2018 | 8 | |
| **264** | Otero, 2016 | 8 | |
| **174** | Page-Shipp, 2018 | 8 | |
| **178** | Ramos, 2013 | 8 | |
| **251** | Vinhas, 2017 | 8 | |
| **76** | Baliashvili, 2016 | 9 | |
| **82** | Chamie, 2015 | 9 | |
| **291** | Dair, 2014 | 9 | |
| **295** | Gazetta, 2006 | 9 | |
| **296** | Grandjean, 2011 | 9 | |
| **217** | Huang, 2014 | 9 | |
| **255** | Jaganath, 2013 | 9 | |
| **73** | Javaid, 2016 | 9 | |
| **101** | Jones-López, 2015 | 9 | |
| **207** | Khatana, 2017 | 9 | |
| **238** | Lima, 2013 | 9 | |
| **303** | Maciel, 2009 | 9 | |
| **275** | Martinez, 2016 | 9 | |
| **212** | Rutherford, 2012 | 9 | |
| **105** | Tadesse, 2016 | 9 | |
| **167** | Triasih, 2015 | 9 | |
| **248** | Ustero, 2017 | 9 | |
| **62** | Adeyekun, 2010 | 10 | 174 adults, comprising fresh university students, newly employed staff and patients referred for Mantoux test on account of contract tracing for PTB, were recruited into the study. Study results do not differentiate each group. |

| | | | |
|---|---|---|---|
| **8** | Amenuvegbe, 2016 | 10 | Descriptive study to identify possible factors contributing to low TB case detection in the district. Interviews with contacts and community health workers. |
| **74** | Aye, 2018 | 10 | Study staff visited the household of TB index patients to screen contacts + extended the screening process to the people who were living in surrounding houses. No clear definition of "surrounding houses". I am assuming these are casual contacts. No stratified data just for household contacts. |
| **328** | Blok, 2015 | 10 | Study with data from different TB Reach studies. We sent an e-mail to the main author to ensure we do not include duplicate data in our analysis. No answer received so far. |
| **107** | Boonthanapat, 2019 | 10 | Retrospective analysis. No CI conducted: authors retrieved the line listing of MDR-TB patients in the hospital between October 2012 and September 2015. All cases were interviewed to identify their contacts and their medical records were reviewed for history of TB screening. |
| **168** | Deery, 2014 | 10 | It is not possible to extract just data for contacts of newly diagnosed TB cases. Five types of index cases were included: newly diagnosed TB cases, newly diagnosed HIV cases, individuals lost to anti-tuberculosis treatment and individuals lost to pre-antiretroviral therapy (pre-ART) care or ART. |

| | | | |
|---|---|---|---|
| **45** | Ekwueme, 2014 | 10 | Index cases diagnosed at different time-points and no information to properly stratify data per period. |
| **29** | Guerra, 2019 | 10 | Study in prison. Prisoners were screened and regarding to the contacts, none of the inmates that share the cell with a TB case (high priority contact) was diagnosed with active TB during the study. |
| **329** | Hanan, 2006 | 10 | Full text not found. |
| **271** | Iqbal, 2013 | 10 | 1-)Just one to 2 contacts per index were recruited to this study; 2-) The duration of contact is unclear; 3-) The final outcome is unclear (numerator). |
| **94** | James, 2017 | 10 | Study compares 3 ACF strategies. For CI they screened household contacts and neighborhood contacts. No clear definition of neighborhood contacts to allow classification as close contact or casual contact. Study provides just the total number of contacts screened. No data just for household contacts. |
| **154** | Kasaie, 2014 | 10 | No CI data: use of an agent based simulation model to explore the dynamics of TB transmission. |
| **235** | Khalilzadeh, 2006 | 10 | Study design is prospective (as reported). However, study does not provide clear information on how data was collected; and it does not provide information to differentiate co-prevalent to incident TB cases among contacts. |

| | | | |
|---|---|---|---|
| **83** | Lebina, 2015 | 10 | Schoolchildren were tested for TB infection QFT-GIT and for positive cases contact investigation was performed on households. Contact tracing did not start from a TB index case. |
| **124** | Lung, 2019 | 10 | Economic evaluation of the ACT2 trial, included as Fox, 2018. |
| **267** | Mutsvangwa, 2010 | 10 | Just tested for TB infection. No information regarding progression to active TB. |
| **144** | Mwansa-Kambafwile, 2013 | 10 | Unsure about classification of contacts (if household contact, close or casual). This is a study to evaluate the effectiveness and acceptability of a paper slip method for TB contact tracing. 16% of the TB contacts did not live with the person who gave them the slip. Of these, the majority (60%) had daily contact with this person. |
| **260** | Nakaoka, 2006 | 10 | Contacts screening occurred >or=12 weeks after diagnosis of the index case. The mean duration between their initial TB diagnosis and follow-up household visits was 54 weeks (range 27-88 weeks). There is no stratified data per year for the incident cases. |
| **102** | Ohene, 2017 | 10 | No definitions of contacts used. Not possible to know if the study had only household contacts and close contacts. |
| **265** | Oliveira, 2017 | 10 | Retrospective study: the strategy used to examine the contacts, the tests performed, and the number of cases of active and latent disease detected among the examined contacts could not be evaluated using the database. |

| | | | |
|---|---|---|---|
| **81** | Ratovoson, 2014 | 10 | Contact investigation was conducted around schoolchildren and their close contacts, not around a confirmed TB index case. The aim of this study was to evaluate the utility of the tuberculin skin test (TST) in first-year schoolchildren as a means of increasing the number of tuberculosis cases detected through the screening of close contacts. |
| **120** | Shah, 2017 | 10 | Cost-effectiveness analysis. Just economic evaluation. No real CI data presented. |
| **34** | Thanh, 2014 | 10 | Not eligible. Retrospective, interviewed household contacts of index cases registered for treatment 2 years before. Do not mention if checked information with TB registries. |
| **68** | Van Wyk, 2012 | 10 | CI of just one household. |
| **84** | Yadav, 2014 | 10 | Cost-effectiveness analysis of ACF strategy. It is nor clear if conducted systematic evaluation of contacts. Not clear if CI. |
| **150** | Yassin, 2013 | 10 | Just evaluation for TB infection. No further evaluation for active TB. |
| **128** | Dierberg, 2016 | 11 | |
| **133** | Gonza´lez-Ochoa, 2009 | 11 | |
| **161** | Jordán Severo, 2008 | 11 | |
| **132** | Krauss, 2015 | 11 | |
| **123** | Li, 2017 | 11 | |
| **137** | Moyo, 2011 | 11 | |
| **202** | Naila, 2013 | 11 | |
| **320** | No author, 2010 | 11 | |
| **184** | Prasad, 2016 | 11 | |
| **136** | Pronyk, 2007 | 11 | |
| **214** | Shivaramakrishna, 2014 | 11 | |

| | | |
|---|---|---|
| **109** | Zhang, 2011 | 11 |

**Exclusion reasons:** 1) High-income country; 2) Abstracts, editorials, correspondence; 3) Case studies or outbreak reports; 4) Only contacts with active TB (no data to calculate yield); 5) Total number of contacts screened not reported (no data to calculate yield); 6) No data do calculate yield (other); 7) No systematic evaluation of contacts; 8) Retrospective study with no data for co-prevalent cases; 9) Duplicated data; 10) Other; 11) No contact investigation conducted.

**Note:** The reference IDs do not follow a chronological order because these numbers reflect the original ID that what assigned to each one of the 329 studies that had full-text screened.
