# Supplementary material for: The yield of tuberculosis contact investigation in low- and middle-income settings: a systematic review and meta-analysis
Source: BMC Infect Dis. 2021 Sep 27;21:1011. doi: 10.1186/s12879-021-06609-3 (PMC8474777; doi:10.1186/s12879-021-06609-3)
Supplement: Supplementary file 4 — Additional file 4: File S1. Adapted Risk of Bias Assessment Tool. [file 12879_2021_6609_MOESM4_ESM.pdf]

S1 File. Adapted Risk of Bias Assessment Tool

1) **Was some form of random selection used to select the sample, OR, was a census undertaken?**
    a) A census was undertaken with an attempt to include all index cases and TB contacts in a specific setting. The answer is: Yes (Low risk).
    b) A census was NOT undertaken. However, the sample was selected using simple random sampling. The answer is: Yes (Low risk).
    c) A census was NOT undertaken, and no random sampling was used. The answer is: No (High risk).
    d) Not reported. The answer is: No (High risk)

2) **Was the likelihood of contacts non-response bias minimal (considering just the initiation of CI, not the whole follow-up period)?**
    a) Yes, participation of contacts was reasonable (> or = 75%). The answer is Yes (Low risk).
    b) No, participation rate was < 75%. The answer is no (High risk).
    c) Not reported. The answer is: No (High risk)

3) **Was the likelihood of index case non-response bias minimal?**
    a) Yes, participation of index cases was reasonable (> or = 75%). The answer is Yes (Low risk).
    b) No, participation rate was < 75%. The answer is no (High risk).
    c) Not reported. The answer is: No (High risk)

4) **Were data collected directly from the contacts (as opposed to a proxy)?**
    a) Data was collected directly from all contacts. The answer is: Yes (Low risk).
    b) In most of the cases, data was collected directly from contacts. The answer is: Yes (Low risk).
    c) Data was collected from a proxy (such as parents in the case of children or through the index case) The answer is: No (High risk).
    d) Retrospective study. The answer is: No (High risk).
    e) Not reported. The answer is: No (High risk).

5) **Was an acceptable case definition used in the study**?
    a) The study used the same definitions proposed by the contact investigation guidelines for index case and TB contacts (household and/or close contact). The answer is: Yes (Low risk).
    b) The study did not use the same definitions proposed by the contact investigation guidelines for index case and TB contacts (household and/or close contact). The answer is: No (High risk).
    c) Not reported. The answer is: No (High risk).

6) **Was an appropriate initial screening of contacts done?**
    a) A screening tool was used for the initial screening of contacts. The answer is: Yes (Low risk).
    b) No screening tool was used for the initial screening of contacts. The answer is: No (High risk).
    c) Not reported. The answer is: No (High risk).

7) **Was the same mode of data collection and investigation used for all TB contacts?**
    a) The same procedures were used for all TB contacts in the study. The answer is Yes (Low Risk).
    b) Different procedures were used to collect data from TB contacts. The answer is No (High risk).
    c) Not reported. The answer is: No (High risk).

Table 1. Comparison between the questions of the original tool to the adapted tool.

Questions proposed by Hoy D. et al.[26]	Decision	New question (if adapted)	Comments

Was the study's target population a close representation of the national population in relation to relevant variables, e.g. age, sex, occupation?	Excluded	N/A	Not applicable, just for prevalence studies.
Was the sampling frame a true or close representation of the target population?	Excluded	N/A	Not applicable, just for prevalence studies.
Was some form of random selection used to select the sample, OR, was a census undertaken?	Kept as the original	N/A	N/A
Was the likelihood of non-response bias minimal?	Adapted (broken in 2 questions, one for index cases and another one for contacts)	Was the likelihood of contacts non-response bias minimal (considering just the initiation of CI, not the whole follow-up period)? / Was the likelihood of index case non-response bias minimal?	N/A
Were data collected directly from the subjects (as opposed to a proxy)?	Kept as the original	N/A	N/A
Was an acceptable case definition used in the study?	Kept as the original	N/A	N/A
Was the study instrument that measured the parameter of interest shown to have reliability and validity (if necessary)?	Adapted	Was an appropriate initial screening of contacts done?	N/A
Was the same mode of data collection used for all subjects?	Adapted	Was the same mode of data collection and investigation used for all TB contacts?	N/A
Was the length of the shortest prevalence period for the parameter of interest appropriate?	Excluded	N/A	We decided to exclude this question instead of adapting for the length of the follow-up period for screening of contacts, because many included studies were published as primary reports, so we could not consider short follow-up periods as as risk of bias.
Were the numerator (s) and denominator(s) for the parameter of interest appropriate?	Excluded	N/A	Question excluded because only studies which provided appropriate data for the numerator and denominator in order to calculate the yield of CI were included.
