# Supplementary material for: The yield of tuberculosis contact investigation in low- and middle-income settings: a systematic review and meta-analysis
Source: BMC Infect Dis. 2021 Sep 27;21:1011. doi: 10.1186/s12879-021-06609-3 (PMC8474777; doi:10.1186/s12879-021-06609-3)
Supplement: Supplementary file 5 — Additional file 5: Table S4. List of the 110 citations included in this systematic review. [file 12879_2021_6609_MOESM5_ESM.pdf]

S4 Table. List of the 110 citations included in this systematic review.

Reference ID	Author, Year	Country	Start Date of data collection	Contributed to yield all TB?	Contributed to yield confirmed TB?	Contributed to yield LTBI?
**1**	Abu-Taleb, 2011	Egypt	2008			X
**3**	Aibana, 2016	Peru	2009	X		X
**194**	Aida, 2012	Egypt	2009	X		X
**6**	Aldhhubhani, 2013	Yemen	2008			X
**7**	Amanullah, 2014	Pakistan	2008	X	X	X
**10**	Armstrong-Hough, 2017	Uganda	2015	X	X	
**11**	Assefa, 2015	Ethiopia	2013	X	X	
**35**	Bakir, 2008	Turkey	2002			
**75**	Baliashvili, 2018	Georgia	2012	X		X
**117**	Batra, 2012	Pakistan	2008	X		
**215**	Becerra, 2011	Peru	Unknown	X		
**204**	Becerra, 2013	Peru	1996	X		
**139**	Beyanga, 2018	Tanzania	2016	X	X	
**263**	Birungi, 2018	Rwanda	2015	X		
**165**	Bonnet, 2017	Uganda	2012	X	X	X
**72**	Cailleaux-Cezar, 2009	Brazil	2000	X		X
**182**	Cavalcante, 2010	Brazil	2000	X		X
**116**	Chakhaia, 2014	Georgia	2010	X	X	X
**65**	Chamie, 2018	Uganda	2012	X	X	
**268**	Chheng, 2015	Uganda	2002	X	X	X
**186**	Dahiwale, 2010	India	2007	X		X
**15**	Datiko, 2017	Ethiopia	2011	X	X	

**13**	Datta, 2017	Peru	2006	X	X	X
**187**	Dayal, 2018	India	2015	X		X
**42**	Diatta, 2007	Senegal	2003	X	X	X
**262**	Eang, 2012	Cambodia	2005	X	X	
**152**	Egere, 2017	Gambia	2012	X	X	X
**179**	Fox, 2012	Vietnam	2009	X	X	
**205**	Fox, 2017	Vietnam	2014	X	X	X
**125**	Fox, 2018	Vietnam	2010	X		
**131**	Garie, 2011	Ethiopia	2007	X		X
**279**	Gashu, 2016	Ethiopia	2014			
**259**	Gomes, 2011	Guinea-Bissau	2005	X		X
**322**	Guo, 2012	China	Unknown	X	X	
**269**	Guo, 2019	China	2010	X	X	
**193**	Gupta, 2016	India	2013	X	X	
**253**	Habte, 2016	Ethiopia	2013	X	X	
**69**	Hall, 2015	Timor-Leste	2013	X	X	
**171**	Hill, 2007	Gambia	Unknown	X	X	
**135**	Hiruy, 2018	Ethiopia	Unknown	X	X	
**216**	Hoang, 2019	Vietnam	2013	X	X	
**278**	Hosten, 2018	Jordan	2014	X	X	X
**254**	Htet, 2018	Myanmar	2015			
**141**	Hu, 2012	China	2009	X		X
**206**	Jackson-Sillaha, 2007	Gambia	2002	X	X	X
**327**	Jafari, 2016	Iran	Unknown			
**240**	Javaid, 2016	Pakistan	2012	X	X	
**241**	Jerene, 2015	Ethiopia	2013	X	X	
**219**	Jia, 2014	China	2008	X	X	
**190**	Jiang, 2018	China	2009	X		

**30**	Jones-Lopez, 2013	Uganda	2009	X	X	X
**261**	Karamagi, 2018	Uganda	2016	X	X	
**115**	Khanal, 2016	Nepal	2013	X	X	
**208**	Khatana, 2019	India	2014	X	X	
**52**	Kilicaslan, 2009	Turkey	1997	X		
**134**	Kliner, 2013	Eswatini	2011	X		
**100**	Kruk, 2008	South Africa	2004	X		
**210**	Lala, 2015	South Africa	2010	X	X	
**227**	Laniado-Laborin, 2014	Mexico	2011			X
**48**	Lin, 2008	China	2006	X		X
**257**	Little, 2018	South Africa	2013	X	X	
**258**	Lu, 2018	China	2011			X
**224**	Machado Leyva, 2007	Cuba	1997			
**245**	Maciel, 2009	Brazil	2003	X	X	
**4**	Mandalakas, 2017	Eswatini	2013	X	X	
**274**	Martinez, 2018	Uganda	1995	X	X	X
**118**	Masur, 2017	Haiti	2013	X	X	
**142**	McAllister, 2017	Indonesia	Unknown			
**180**	Mensah, 2017	Ghana	2012			X
**191**	Muyoyeta, 2017	Zambia	2013	X	X	
**189**	Narasimhan, 2017	India	2010	X	X	X
**181**	Nguyen, 2009	Lao PDR	2006	X	X	X
**95**	Ntinginya, 2012	Tanzania	2011			
**140**	Oshi, 2017	Nigeria	2013	X	X	
**237**	Pérez-Porcuna, 2012	Brazil	2008	X	X	
**175**	Pothukuchi, 2011	India	2008			
**244**	Puryear, 2013	Botswana	2009	X	X	
**90**	Qadeer, 2017	Pakistan	2013	X	X	
**56**	Qader, 2017	Afghanistan	2013	X		

**77**	Rekha, 2009	India	2008			
**53**	Rekha, 2013	India	2009			
**211**	Rutherford, 2012	Indonesia	Unknown	X		X
**54**	Sanaie, 2016	Afghanistan	2010	X	X	
**266**	Seddon, 2013	South Africa	2010	X	X	X
**49**	Shah, 2013	Pakistan	2010	X	X	
**281**	Shamaei, 2018	Iran	2011	X	X	X
**249**	Shapiro, 2012	South Africa	2009	X	X	
**213**	Sia, 2010	Philippines	2001	X	X	X
**192**	Sinfield, 2013	Malawi	2003	X		X
**270**	Singh, 2012	India	2006	X	X	X
**276**	Singh, 2013	India	2007	X	X	X
**23**	Singla, 2011	India	2010	X	X	
**89**	Stein, 2018	Uganda	2002	X		X
**87**	Sulis, 2018	Burkina Faso	2016	X		
**55**	Swindells, 2018	Multi-country	2015	X	X	
**138**	Thind, 2012	South Africa	2009	X		
**273**	Tieu, 2014	Thailand	2009			
**243**	Triasih, 2015	Indonesia	2010	X		X
**330**	van Schalkwyk, 2014	South Africa	2010			
**121**	van Zyl, 2006	South Africa	1996	X		X
**250**	Vella, 2011	South Africa	2005	X	X	
**272**	Verhagen, 2014	Venezuela	2010	X	X	X
**80**	Villegas, 2014	Colombia	2005			
**151**	Volkmann, 2016	Kenya	2014			
**43**	Whalen, 2011	Uganda	Unknown		X	X
**325**	Xu, 2008	China	Unknown			
**93**	Yuhara, 2013	Brazil	2006	X		

**222**	Zawedde-Muyanja, 2018	Uganda	2015	X	
**46**	Zelner, 2014	Peru	2009	X	
**246**	Zhang, 2011	China	2007	X	X

Note: The reference IDs do not follow a chronological order because these numbers reflect the original ID that what assigned to each one of the 329 studies that had full-text screened.
