# Supplementary material for: The yield of tuberculosis contact investigation in low- and middle-income settings: a systematic review and meta-analysis
Source: BMC Infect Dis. 2021 Sep 27;21:1011. doi: 10.1186/s12879-021-06609-3 (PMC8474777; doi:10.1186/s12879-021-06609-3)
Supplement: Supplementary file 6 — Additional file 6: Table S5. Data on Risk of Bias for each study. [file 12879_2021_6609_MOESM6_ESM.pdf]

S5 Table. Data on Risk of Bias for each study.

Author, year	#1	#2	#3	#4	#5	#6	#7	Summary
Abu-Taleb, 2011	Low	Low	Low	Low	Low	Low	Low	Low
Aibana, 2016	Low	High	High	Low	High	Low	Low	Moderate
Mandalakas, 2017	Low	High	High	Low	High	Low	Low	Moderate
Aldhhubhani, 2013	High	Low	High	Low	Low	Low	Low	Moderate
Amanullah, 2014	Low	Low	High	Low	Low	Low	High	Moderate
Armstrong-Hough, 2017	Low	High	High	Low	Low	Low	Low	Moderate
Assefa, 2015	Low	High	High	High	Low	High	Low	Moderate
Datta, 2017	Low	High	High	Low	Low	Low	Low	Low
Datiko, 2017	Low	High	High	Low	Low	Low	Low	Low
Singla, 2011	Low	High	High	Low	Low	High	High	Moderate
Jones-Lopez, 2013	Low	Low	Low	Low	Low	High	Low	High
Bakir, 2008	Low	Low	Low	Low	Low	Low	Low	Low
Diatta, 2007	Low	High	High	Low	Low	Low	Low	High
Whalen, 2011	Low	High	High	Low	High	Low	Low	Low
Zelner, 2014	Low	High	High	Low	High	Low	Low	Low
Lin, 2008	Low	Low	High	Low	Low	Low	Low	Moderate
Shah, 2013	Low	High	Low	Low	Low	Low	Low	Moderate
Kilicaslan, 2009	Low	Low	High	High	Low	Low	Low	Moderate
Rekha, 2013	Low	High	Low	Low	Low	Low	Low	Moderate
Sanaie, 2016	Low	High	High	Low	Low	Low	Low	Moderate
Swindells, 2018	Low	High	High	High	Low	Low	Low	Moderate
Qader, 2017	Low	High	High	Low	Low	Low	Low	Moderate
Chamie, 2018	Low	High	Low	Low	Low	Low	Low	Moderate
Hall, 2015	Low	High	Low	Low	Low	Low	Low	Moderate
Cailleaux-Cezar, 2009	Low	High	Low	Low	Low	Low	Low	Moderate
Baliashvili, 2018	Low	High	High	High	Low	High	Low	Moderate
Rekha, 2009	Low	High	High	High	High	High	High	High

Villegas, 2014	Low	Low	Low	Low	Low	Low	High	Moderate
Sulis, 2018	Low	Low	High	Low	High	Low	Low	Moderate
Stein, 2018	Low	Low	Low	Low	Low	Low	Low	Low
Qadeer, 2017	Low	High	High	High	Low	Low	Low	Moderate
Yuhara, 2013	Low	High	High	High	Low	High	High	High
Ntinginya, 2012	Low	High	Low	Low	Low	Low	Low	Moderate
Kruk, 2008	Low	Low	High	Low	Low	Low	Low	Moderate
Khanal, 2016	Low	High	High	Low	Low	Low	Low	Moderate
Chakhaia, 2014	Low	High	High	High	Low	Low	Low	Moderate
Batra, 2012	Low	High	High	High	Low	Low	Low	Moderate
Masur, 2017	Low	High	Low	Low	High	Low	Low	Moderate
van Zyl, 2006	Low	High	High	High	Low	Low	Low	Moderate
Fox, 2018	Low	Low	Low	Low	Low	Low	Low	Low
Garie, 2011	Low	High	High	Low	High	Low	Low	Moderate
Kliner, 2013	Low	High	High	Low	Low	Low	Low	Moderate
Hiruy, 2018	Low	Low	Low	Low	Low	Low	Low	Moderate
Thind, 2012	Low	High	High	High	Low	Low	High	High
Beyanga, 2018	Low	High	High	High	Low	Low	Low	High
Oshi, 2017	Low	High	High	High	High	Low	Low	High
Hu, 2012	Low	Low	High	High	Low	Low	Low	Moderate
McAllister, 2017	Low	Low	Low	Low	Low	Low	Low	Moderate
Volkmann, 2016	Low	Low	Low	Low	High	Low	Low	Moderate
Egere, 2017	Low	Low	Low	Low	Low	Low	Low	Moderate
Bonnet, 2017	Low	Low	High	Low	Low	Low	Low	Moderate
Hill, 2007	Low	Low	High	Low	High	High	Low	High
Pothukuchi, 2011	Low	High	Low	Low	Low	Low	Low	Moderate
Fox, 2012	High	Low	High	Low	Low	Low	Low	Moderate
Mensah, 2017	Low	Low	High	Low	Low	High	Low	Moderate
Nguyen, 2009	Low	Low	Low	Low	Low	Low	Low	Low
Cavalcante, 2010	Low	High	High	Low	Low	Low	Low	Moderate
Dahiwale, 2010	Low	High	High	Low	High	Low	Low	Moderate

Dayal, 2018	Low	High	High	Low	Low	Low	Low	Moderate
Narasimhan, 2017	Low	High	High	Low	Low	Low	Low	Moderate
Jiang, 2018	Low	High	Low	Low	High	Low	Low	Moderate
Muyoyeta, 2017	Low	High	Low	Low	High	Low	Low	Moderate
Sinfield, 2013	Low	High	High	Low	High	Low	Low	Moderate
Gupta, 2016	Low	High	Low	Low	Low	Low	Low	Moderate
Aida, 2012	Low	Low	High	Low	Low	Low	Low	Moderate
Becerra, 2013	Low	High	High	High	Low	Low	High	High
Fox, 2017	Low	High	High	Low	Low	Low	Low	Moderate
Jackson-Sillaha, 2007	Low	High	High	Low	Low	Low	Low	Moderate
Khatana, 2019	High	Low	High	Low	High	Low	Low	Moderate
Lala, 2015	Low	High	High	Low	Low	Low	Low	Moderate
Rutherford, 2012	Low	High	High	Low	Low	Low	Low	Moderate
Sia, 2010	Low	Low	High	High	Low	Low	Low	Moderate
Becerra, 2011	Low	High	High	High	High	High	High	High
Hoang, 2019	Low	Low	High	Low	Low	Low	Low	Moderate
Jia, 2014	Low	Low	High	Low	Low	Low	Low	Moderate
Zawedde-Muyanja, 2018	High	High	High	Low	High	Low	Low	High
Machado Leyva, 2007	Low	High	High	Low	High	High	Low	High
Laniado-Laborin, 2014	Low	High	High	Low	High	Low	Low	Moderate
Pérez-Porcuna, 2012	High	High	High	Low	High	Low	Low	Moderate
Javaid, 2016	Low	High	Low	Low	Low	Low	Low	Moderate
Jerene, 2015	Low	Low	High	Low	Low	Low	Low	Low
Triasih	Low	High	High	Low	Low	Low	Low	Moderate
Puryear, 2013	Low	High	High	Low	Low	Low	Low	High
Maciel, 2009	Low	High	Low	Low	High	Low	Low	Moderate
Zhang, 2011	Low	High	High	Low	High	Low	Low	Moderate
Shapiro, 2012	Low	High	Low	Low	Low	Low	Low	Moderate

Vella, 2011	Low	High	High	Low	High	Low	Low	Moderate
Habte, 2016	Low	Low	Low	Low	Low	Low	Low	Moderate
Htet, 2018	Low	High	High	Low	High	Low	High	High
Little, 2018	Low	High	Low	Low	Low	Low	Low	Moderate
Lu, 2018	Low	Low	Low	Low	Low	High	Low	Low
Gomes, 2011	Low	High	High	Low	High	Low	Low	Moderate
Karamagi, 2018	Low	High	High	Low	High	Low	Low	Moderate
Eang, 2012	Low	High	High	Low	High	Low	Low	Moderate
Birungi, 2018	Low	High	Low	Low	Low	Low	Low	Moderate
Seddon, 2013	Low	Low	Low	Low	Low	Low	Low	Low
Chheng, 2015	High	Low	High	Low	Low	Low	Low	Moderate
Guo, 2019	Low	High	High	High	High	Low	High	High
Singh, 2012	Low	High	Low	Low	Low	Low	Low	Moderate
Verhagen, 2014	Low	Low	Low	Low	High	High	Low	Moderate
Tieu, 2014	Low	High	High	Low	High	Low	Low	High
Martinez, 2018	Low	High	High	Low	Low	Low	Low	Moderate
Singh, 2013	Low	Low	Low	Low	Low	Low	Low	Low
Hosten, 2018	Low	High	High	High	Low	High	Low	High
Gashu, 2016	Low	Low	High	Low	Low	Low	Low	Moderate
Shamaei, 2018	Low	High	High	Low	Low	Low	Low	Moderate
Guo, 2012	Low	Low	Low	Low	Low	Low	Low	Low
Xu, 2008	Low	Low	Low	Low	Low	Low	Low	Low
Jafari, 2016	Low	High	High	High	Low	Low	Low	High
van Schalkwyk, 2014	Low	High	Low	Low	Low	Low	Low	Moderate

Notes: The numbers on the title of each column refer to the question number as presented on the adapted Risk of Bias Assessment Tool; the summary rating is the rater's subjective judgement given the answers for the preceding questions.

Questions:

1) Was some form of random selection used to select the sample, OR, was a census undertaken? a) A census was undertaken with an attempt to include all index cases and TB contacts in a specific setting. The answer is: Yes (Low risk).
b) A census was NOT undertaken. However, the sample was selected using simple random sampling. The answer is: Yes (Low risk).

c) A census was NOT undertaken, and no random sampling was used. The answer is: No (High risk).
d) Not reported. The answer is: No (High risk)

2) Was the likelihood of contacts non-response bias minimal (considering just the initiation of CI, not the whole follow-up period)? a) Yes, participation of contacts was reasonable (> or = 75%). The answer is Yes (Low risk).
b) No, participation rate was < 75%. The answer is no (High risk).
c) Not reported. The answer is: No (High risk)

3) Was the likelihood of index case non-response bias minimal? a) Yes, participation of index cases was reasonable (> or = 75%). The answer is Yes (Low risk).
b) No, participation rate was < 75%. The answer is no (High risk).
c) Not reported. The answer is: No (High risk)

4) Were data collected directly from the contacts (as opposed to a proxy)? a) Data was collected directly from all contacts. The answer is: Yes (Low risk).
b) In most of the cases, data was collected directly from contacts. The answer is: Yes (Low risk).
c) Data was collected from a proxy (such as parents in the case of children or through the index case) The answer is: No (High risk).
d) Retrospective study. The answer is: No (High risk).
e) Not reported. The answer is: No (High risk).

5) Was an acceptable case definition used in the study? a) The study used the same definitions proposed by the contact investigation guidelines for index case and TB contacts (household and/or close contact). The answer is: Yes (Low risk).
b) The study did not use the same definitions proposed by the contact investigation guidelines for index case and TB contacts (household and/or close contact). The answer is: No (High risk).
c) Not reported. The answer is: No (High risk).

6) Was an appropriate initial screening of contacts done? a) A screening tool was used for the initial screening of contacts. The answer is: Yes (Low risk).
b) No screening tool was used for the initial screening of contacts. The answer is: No (High risk).
c) Not reported. The answer is: No (High risk).

7) Was the same mode of data collection and investigation used for all TB contacts? a) The same procedures were used for all TB contacts in the study. The answer is Yes (Low Risk).
b) Different procedures were used to collect data from TB contacts. The answer is No (High risk).
c) Not reported. The answer is: No (High risk).
