# Supplementary material for: The yield of tuberculosis contact investigation in low- and middle-income settings: a systematic review and meta-analysis
Source: BMC Infect Dis. 2021 Sep 27;21:1011. doi: 10.1186/s12879-021-06609-3 (PMC8474777; doi:10.1186/s12879-021-06609-3)
Supplement: Supplementary file 7 — Additional file 7: File S2. Sensitivity Analysis. [file 12879_2021_6609_MOESM7_ESM.pdf]

S2 File. Sensitivity Analysis

Table 1. Sensitivity analysis by removing outliers.

	n	Pooled yield for all active TB	n	Pooled yield for confirmed active TB	n	Pooled yield for LTBI
Initial yields calculated	89	2.87% (2.61-3.14, I^2 97.79%)	59	2.04% (1.77-2.31, I^2 98.06%)	42	43.83% (38.11-49.55, I^2 99.36%)
Sensitivity analysis (outliers removed)	84	2.58% (2.33-2.84, I^2 97.69%)	55	1.93% (1.66-2.20, I^2 98.14%)	36	43.47% (38.64-48.30, I^2 98.83%)

*All pooled yield results are presented with a 95% confidence interval and I^2 for heterogeneity.

Table 2. Sensitivity analysis by removing studies with restrictions on the target population.

	n	Pooled yield for all active TB	n	Pooled yield for confirmed active TB	n	Pooled yield for LTBI
Initial yields calculated	89	2.87% (2.61-3.14, I^2 97.79%)	59	2.04% (1.77-2.31, I^2 98.06%)	42	43.83% (38.11-49.55, I^2 99.36%)
Sensitivity analysis	54	2.39% (2.09-2.69, I^2 98.20%)	40	1.92% (1.61-2.24, I^2 98.54%)	27	47.74% (41.15-54.32, I^2 99.28%)

*All pooled yield results are presented with a 95% confidence interval and I^2 for heterogeneity.

Table 3. Sensitivity analysis by risk of bias classification.

	n	Pooled yield for all active TB	n	Pooled yield for confirmed active TB	n	Pooled yield for LTBI
All risk of bias classification	89	2.87% (2.61-3.14, I^2 97.79%)	59	2.04% (1.77-2.31, I^2 98.06%)	42	43.83% (38.11-49.55, I^2 99.36%)
Just studies classified as low or medium risk of bias	76	3.14% (2.84-3.44, I^2 97.86%)	51	2.23% (1.91-2.55, I^2 98.28%)	39	43.38% (37.46-49.30, I^2 99.38%)
Just studies classified as low risk of bias	10	2.01% (1.47-2.55, I^2 98.13%)	8	1.16% (0.78-1.55, I^2 97.40%)	8	47.83% (32.60-63.05, I^2 99.46%)

*All pooled yield results are presented with a 95% confidence interval and I^2 for heterogeneity.
